# Prevalence of self-reported dry eye symptoms after refractive surgery among Saudi adults: A cross-sectional survey

**DOI:** 10.1371/journal.pone.0361098

**Published:** 2026-10-29

**Authors:** Mahmood J. Showail, Abubakr S. Alzwaihri, Eid A. Almasoudi, Hassan A. Ahmed, Mohammed K. Alghamdi, Mohammed A. Taha, Waseem A. Aalam

**Affiliations:** 1 Department of Ophthalmology, Faculty of Medicine, King Abdulaziz University, Jeddah, Saudi Arabia; 2 Department of Ophthalmology, King Abdulaziz University Hospital, King Abdulaziz University, Jeddah, Saudi Arabia; 3 Department of Ophthalmology, Jeddah Eye Hospital, Jeddah, Saudi Arabia; 4 Department of Otolaryngology Head & Neck Surgery, Mouwasat Hospital, Dammam, Saudi Arabia; 5 Department of Orthopedic, King Salman Armed Forces Hospital, Tabuk, Saudi Arabia; 6 Department of Dermatology, King Fahad hospital, Jeddah, Saudi Arabia; 7 King Saud Bin Abdulaziz University for Health Sciences, Riyadh, Saudi Arabia; 8 Department of Surgery, College of Medicine, University of Jeddah, Jeddah, Saudi Arabia; University of Missouri-Columbia, UNITED STATES OF AMERICA

## Abstract

Refractive surgery corrects vision using different corneal techniques; however, dry eye disease (DED) is one of its commonest side effects. This descriptive survey questionnaire-based study aimed to evaluate prevalence and factors associated with dry eye symptoms after refractive surgery among survey respondents using ocular surface disease index (OSDI) questionnaire in Saudi Arabia. Four hundred and thirty nine participants were included. Dry eye OSDI scores compared across different epidemiological risk factors, focusing on refractive surgery type and postoperative duration. Photorefractive keratectomy (PRK), laser-assisted in situ keratomileusis (LASIK) or Femtosecond (FS)-LASIK, and small incision lenticule extraction procedures were performed on 201 (45.79%), 222 (50.56%), and 16 (3.65%) participants, respectively. According to OSDI scores, 391 participants (89.1%) had dry eye symptoms after surgery, with 69.5% classified as severe, 11.4% as moderate, 8.2% as mild, and only 10.9% as normal. Insignificant gender differences were observed; however, OSDI-defined dry eye symptoms prevalence was lower in Eastern Province and in participants with longer post-surgery durations. Participants who underwent PRK reported higher prevalence of OSDI-defined dry eye symptoms versus those underwent LASIK/FS-LASIK. In conclusion, Self-reported OSDI-defined DED were common after refractive surgery, especially after PRK. Females and early post-surgery patients have more severe OSDI-defined dry eye symptoms. Living in a humid area (Eastern Province) may reduce risk of OSDI-defined DED post-surgery.

## Introduction

Refractive surgery is a common elective procedure for vision correction and is considered a safe alternative for people who intend to dispose of glasses and contact lenses [1]. Refractive surgery techniques can be categorized into corneal surface ablation, corneal stromal procedures, and refractive corneal lenticule extraction [1]. Surface ablation procedures involve photorefractive keratectomy (PRK), transepithelial PRK (T-PRK), laser epithelial keratomileusis (LASIK), and epithelial LASIK (Epi-LASIK) [2]. Corneal stromal ablation procedures involve LASIK with a flap created with either a mechanical or femtosecond-based microkeratome (FS-LASIK) [3]. Refractive corneal lenticular extraction procedures involve femtosecond lenticule extraction (FLEx) and small incision lenticule extraction (SMILE) [2]. Dry eye is a common side effect of refractive surgery, despite the high satisfaction rates associated with these procedures [3]. The most prevalent type of postoperative dry eye disease (DED) after ophthalmic surgery is post-LASIK dry eye [4].

Dry eye is a multifactorial illness of the tears and ocular surface that causes symptoms of pain, visual disruption, and tears film instability, with significant corneal surface damage [4,5]. Up to 95% of patients develop dry eyes after refractive surgery, and a percentage of these patients develop chronic eye symptoms, often known as DED [4]. Advanced age, female gender [5], certain medications, contact lens wear, presence of blepharitis, smoking, refractive surgery, extremely hot or cold weather, and low relative humidity are all risk factors for DED [5]. Furthermore, ophthalmic disorders, autoimmune diseases, pain, and musculoskeletal disorders are risk factors for DED [5].

The global prevalence of DED is between 5 and 87.5% in the general population [6,7]. After 50 years of age, the prevalence of DED increases every 5 years, with females having a higher prevalence than males, according to epidemiological studies [8]. DEDs are common in Al-Ahsa, Saudi Arabia, where 32.1% of the population is symptomatic [9].

To the best of our knowledge, no study has estimated the prevalence of dry eye after refractive surgery in the Saudi population. Hence, this study aimed to assess the prevalence of OSDI-defined dry eye symptoms following refractive surgery among survey respondents in the general population of Saudi Arabia and identify associated risk factors.

## Materials and methods

### Study design

This descriptive survey questionnaire-based study conducted among the general population of Saudi Arabia over 6 months from 1/2/2022, to 30/6/2022. An anonymous online self-administered valid dry eye questionnaire (DEQ) (Barbara Caffery) distributed through social media platforms using convenience sampling. Because the survey link was openly shared, the number of individuals who viewed or opened the invitation could not be determined. Therefore, the reported response rate refers only to completed questionnaires. Participants self-reported their history and type of refractive surgery, and no clinical verification or review of medical records was performed. Duplicate submissions were minimized by limiting one response per Google account. The questionnaire contained demographic and clinical characteristics such as sex, age, residence area, smoking status, chronic illness, type of refractive surgery, and time since surgery.

### Study participants

All participants aged >18 years who underwent refractive surgery, lived in Saudi Arabia during the study period, and agreed to participate were enrolled. Patients were excluded from the study if they had existing eye diseases such as glaucoma, keratitis, or severe conjunctivitis; used contact lenses; had autoimmune diseases such as Sjögren’s syndrome or rheumatoid arthritis; or taking medications like antidepressants, antihistamines, or diuretics known to exacerbate dry eye symptoms; or were under 18 years of age.

### Sample size and data collection

The sample size was calculated using Raosoft software. The study required a sample size of 385 participants, determined based on a confidence level of 95% and a margin of error of 5%.

### Ethics approval

This study has received ethical approval from Unit of Biomedical Ethical Research Committee of King Abdulaziz University, Jeddah, Saudi Arabia (Reference #1722, January 25, 2022). The first page of the questionnaire included information on the study objectives and electronic written informed consent to participate before completing the questionnaire. The collected data were stored, kept confidential, and used only for research purposes. Adhering to the ethical principles outlined in the Declaration of Helsinki, every aspect of data collection was conducted with utmost regard for the well-being and rights of the participants.

### Data analysis

Data were analyzed using SPSS version 20 (IBM software package). Categorical variables were presented as frequencies and percentages. Categorical data were compared using the Pearson Chi-Square test. A multivariable logistic regression model was constructed including age, sex, geographic regions, smoking status, chronic diseases, refractive surgery type, and postoperative duration. Odds ratios (ORs) and confidence intervals (CIs) were generated for the side effects. LASIK and FS-LASIK were analyzed together because both procedures share similar surgical principles and because of sample-size considerations. Statistical significance was set at *p* < 0.05.

## Results

### Patient characteristics

The data included responses from (n = 439) participants who complete the questionnaire with response rate (100%). The ocular surface disease index (OSDI) measured to distinguish between healthy participants and those with OSDI-defined dry eye symptoms. The results showed that 391 participants had dry eyes, with the majority (69.5%) having a score of 33–100, 11.4% having a score of 23–32, 8.2% having a score of 13–22, and only 48 (10.9%) having a normal OSDI score. There were 268 (61.04%) females and 171 (38.96%) males, with a female-to-male ratio of 1.57:1. Of the 391 participants with OSDI-defined dry eye symptoms, 243 were female and 148 were male, with no significant difference between them (P > 0.05). Their ages ranged from 18 to more than 50 years, with most study participants aged between 18 and 25 years (n = 223). Most participants were from western Saudi Arabia (n = 125). Only 52 (11.8%) of the study participants were smokers. Sixty-one participants (two healthy participants and 59 with OSDI-defined dry eye symptoms) had one or more chronic conditions. Types of refractive surgery were PRK (n = 201, 45.78%), LASIK/ FS-LASIK (n = 222, 50.56%), and Femto-SMILE (n = 16, 3.65%). The duration since surgery was < 1 year for 161 participants, between 1 and 5 years for 187 participants, and >5 years for 91 participants [Table 1; Figs 1 and 2].

**Table 1:** Demographic and clinical characteristics of the study participants.

Characteristics	Ocular surface disease index (OSDI)	
	Normal participants (OSDI* score 0–12) (n = 48)	Participants with dry eye (n = 391)
**Gender**		
Male (n = 171)	23 (13.5%)	148 (86.5%)
Female (n = 268)	25 (9.3%)	243 (90.7%)
**Age group**		
18–25 years (n = 223)	20 (9.0%)	203 (91.0%)
26–30 years (n = 111)	13 (11.7%)	98 (88.3%)
31–40 years (n = 59)	10 (16.9%)	49 (83.1%)
41–50 years (n = 32)	5 (15.6%)	27 (84.4%)
>50 years (n = 14)	0 (0.0%)	14 (100.0%)
**Residence area**		
Central region (n = 121)	10 (8.3%)	111 (91.7%)
Western region (n = 125)	8 (6.4%)	117 (93.6%)
Eastern region (n = 64)	12 (18.8%)	52 (81.3%)
Northern region (n = 49)	9 (18.4%)	40 (81.6%)
Southern region (n = 80)	9 (11.3%)	71 (88.8%)
**Smoking**		
No (n = 387)	41 (10.6%)	346 (89.4%)
Yes (n = 52)	7 (13.5%)	45 (86.5%)
**Diagnosis of any chronic conditions**		
No (n = 378)	46 (12.2%)	332 (87.8%)
Yes (n = 61)	2 (3.3%)	59 (96.7%)
**Type of refractive surgery**		
Photorefractive keratectomy (PRK) (n = 201)	12 (6.0%)	189 (94.0%)
LASIK or femtosecond laser (n = 222)	33 (14.9%)	189 (85.1%)
Femto-SMILE (n = 16)	3 (18.8%)	13 (81.3%)
**Time since the surgery**		
<1 year (n = 161)	9 (5.6%)	152 (94.4%)
1–5 years (n = 187)	25 (13.4%)	162 (86.6%)
>5 years (n = 91)	14 (15.4%)	77 (84.6%)

LASIK: Laser-assisted in situ keratomileusis, SMILE: Small incision lenticule extraction.

**Fig 1:**
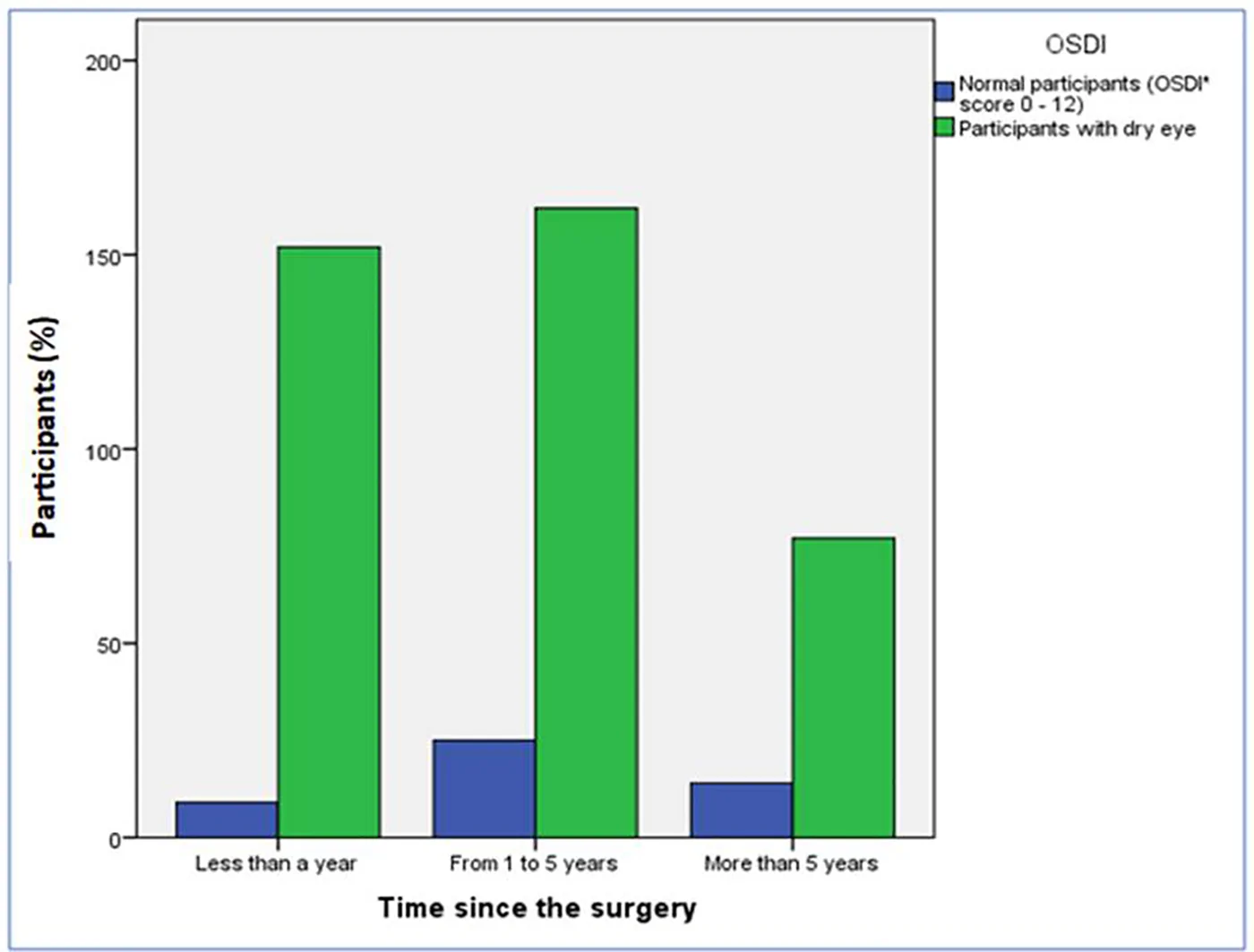
Time since the corrective eye surgery operation among patients with and without dry eye disease. OSDI: Ocular surface disease index.

**Fig 2:**
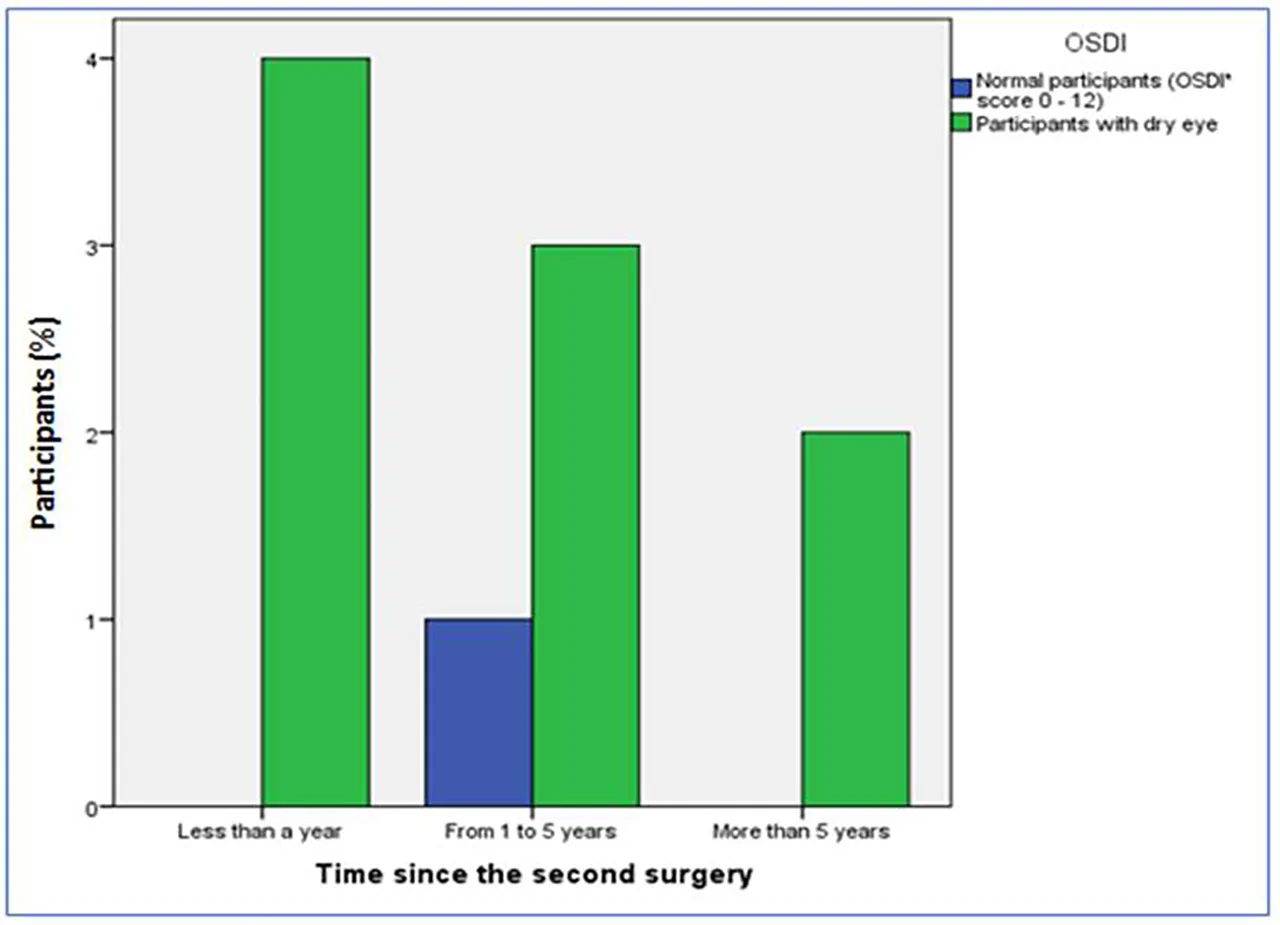
Time since the corrective eye surgery operation among patients with and without dry eye disease. OSDI: Ocular surface disease index.

### Risk factors for dry eye disease

OSDI-defined dry eye symptoms was less prevalent among inhabitants of the Eastern Province of Saudi Arabia than in the central region (OR: 0.390, 95% CI: 0.158–0.962, P = 0.041). OSDI-defined dry eye symptoms was less prevalent among patients who underwent LASIK/FS-LASIK than PRK (OR: 0.364; 95% CI: 0.182–0.726, P = 0.004). OSDI-defined dry eye symptoms was less common among patients with a time since the operation of >5 years and 1–5 years compared to those with an operation time of <1 year (OR: 0.384, 95% CI: 0.174–0.848, P = 0.018 and OR: 0.326, 95% CI: 0.135–0.786, P = 0.013, respectively) [Table 2].

**Table 2:** Multivariable logistic regression analysis of factors associated with OSDI-defined dry eye symptoms.

Factors	P-value	OR	95% CI for OR		Nagelkerke R Square
			Lower	Upper	
**Gender**					
Male		*Ref*			
Female	0.179	1.511	0.827	2.758	0.008
**Age**					
18–25 years		*Ref*			
26–30 years	0.430	0.743	0.355	1.555	0.031
31–40 years	0.082	0.483	0.212	1.097	
41–50 years	0.243	0.532	0.184	1.534	
>50 years	0.999	0.999	–	–	
**Residence area**					
Central region		*Ref*			
Western region	0.575	1.318	0.502	3.459	0.044
Eastern region	**0.041**	0.390	0.158	.962	
Northern region	0.064	0.400	0.152	1.057	
Southern region	0.480	0.711	0.275	1.835	
Smoking (yes)	0.535	0.762	0.322	1.799	0.002
Diagnosis of any chronic conditions (yes)	0.056	4.087	0.966	17.295	0.025
**Type of refractive surgery**					
Photorefractive keratectomy (PRK)		*Ref*			
LASIK or femtosecond laser	**0.004**	0.364	0.182	0.726	0.045
Femto-SMILE	0.068	0.275	0.069	1.098	
**Time since the surgery**					
<1 year		*Ref*			
1–5 years	**0.018**	0.384	0.174	0.848	0.038
>5 years	**0.013**	0.326	0.135	0.786	

OR: Odds ratio, CI: Confidence interval, LASIK: Laser-assisted in situ keratomileusis, SMILE: Small incision lenticule extraction.

## Discussion

Refractive surgery is associated with numerous complications, of which dry eye is the most frequent. The development of blurred vision, visual impairment, pain or burning sensation, dryness, or redness after refractive surgery increases the susceptibility to DED [10]. In this study, the OSDI was used to distinguish between healthy participants and those with OSDI-defined dry eye symptoms among survey respondents. Approximately 89.1% of the respondents experienced postoperative OSDI-defined dry eye symptoms, indicating a substantial prevalence of the condition. According to a study, dry eye symptoms were reported by 95% of patients after refractive surgery, especially during the early postoperative period [11]. Another study aimed to estimate the prevalence and compare outcomes of patients after SMILE and LASIK and reported a high rate of signs and symptoms of DED in both groups [12]. The variations in postoperative ocular surface outcomes between LASIK and lenticule extraction treatments have been further validated by more recent data. Lenticule extraction methods were typically linked to better postoperative dry eye outcomes, according to a 2025 study comparing keratorefractive lenticule extraction surgery with LASIK. This is probably because flap-based treatments cause less damage to the corneal nerve. These results corroborate the increasing amount of research indicating that surgical features unique to a surgery may affect the intensity and duration of postoperative dry eye symptoms [13]. In certain individuals who continue to have postoperative discomfort, tear-conservation techniques may also be useful. In carefully chosen patients, punctal occlusion can enhance symptoms and tear film integrity, according to a recent evidence synthesis assessing punctal plugs for dry eye illness. Although more research is required to ascertain its efficacy specifically in post-refractive populations, this strategy may be especially pertinent for people who have decreased tear production following refractive surgery [14]. A Chinese multicenter study reported that the prevalence of DED after refractive surgery was 41.43% [15]. Similarly, the prevalence of DED in Hong Kong patients after LASIK surgery was 94.8%, 85.4% and 59.4% at 1 day, 1 week and 1 month, respectively [16], which was lower than that in this study. According to recent studies, DED is more common in females, which may be due to the possible association between DED and autoimmune disorders [17]. Moreover, differences in lifestyle, metabolism, hormones, and the body's response to disease may play principal roles in this domain [17]. In a similar methodology study, Shehadeh-Mashor et al. studied the factors associated with the development of DED after refractive surgery in a large database study that extended for approximately 8 years. They concluded that females were more likely to develop DED [3]. Similarly, our results revealed a higher prevalence in females; however, the difference was not statistically significant. This survey demonstrated a low prevalence of dry eye in the elderly group (>50 years) after refractive surgery, which was unexpected. Several studies have reported that DED after refractive surgery is associated with increased age [3,18]. In contrast, two population-based studies reported a higher prevalence of DED among young participants, including high school students, indicating that DED tends to predominate in this age group [19,20]. Age-related changes in tear secretion and increased tear evaporation contribute to the development of DED [6]. Furthermore, the function of the lacrimal gland may increase the risk of developing the disease. A similar report linked DED with inflammation and a decrease in the immune response, which may explain the higher prevalence of the disease in the elderly group.

According to our survey, smoking and chronic diseases were not associated with an increased risk of developing OSDI-defined dry eye symptoms. Smoking toxins can reduce blood flow, cause discomfort, and increase the risk of multiple eye conditions; however, the exact relationship between smoking and DED is not fully understood, and numerous studies have failed to detect a significant relationship [21]. In contrast, Bhutia et al. reported that smoking exposure increases the risk of DED [22]. Further analysis is required to clarify this issue. Systemic diseases, particularly autoimmune and connective tissue disorders, have been linked to DED [23]. However, the most prevalent chronic disorders, such as diabetes mellitus and hypertension, were not reported as potential causative factors, which may have led to the negative relationship observed in our study. Certain environmental factors, such as low humidity and chemical fumes, can increase the risk of developing DED [23]. Participants residing in the Eastern Province reported a lower prevalence of dry eye symptoms. However, environmental humidity was not measured in this study, and this association may reflect other unmeasured factors including recruitment differences, socioeconomic status, clinical practice variation, occupational exposures, or postoperative care.. Another significant cause of dry eye is meibomian gland dysfunction, which can coexist with or exacerbate symptoms following refractive surgery. Both LipiFlow thermal pulsation and intense pulsed light therapy can enhance meibomian gland function and dry eye symptoms, according to recent comparative research, albeit the degree and duration of benefit may vary between treatments. Meibomian gland status was not evaluated in this study, although this mechanism should be taken into account when interpreting chronic postoperative dry eye complaints [24].

DED after refractive surgery has been well described in the literature. DED can occur in up to 48% of patients who undergo LASIK correction surgery; however, the exact prevalence of PRK has not yet been reported [25]. In this study, PRK was the most common procedure associated with DED. PRK is linked to a higher prevalence of dry eye compared to LASIK due to differences in surgical techniques and healing processes. In PRK, the corneal epithelium is removed, exposing underlying nerves and stroma, resulting in more extensive nerve disruption. In contrast, LASIK preserves most of the corneal surface by creating a flap, allowing for faster nerve recovery and shorter duration of dry eye symptoms. PRK also causes greater epithelial disruption, triggering a stronger inflammatory response and delaying epithelial healing, which can intensify dry eye symptoms. LASIK patients generally experience less epithelial damage and reduced inflammation. Early after surgery, PRK patients often report more significant vision fluctuations and foreign body sensations, both linked to pronounced dry eye symptoms. According to Cohen et al., nearly all patients who underwent LASIK ultimately experienced symptoms of DED to varying degrees [26]. A dry eye is the most common postoperative complication of LASIK surgery [26]. Pre-exciting dry eye is the most significant factor in developing DED after LASIK surgery [26,27]. Regarding the time since the operation, the results showed that the longer the time since the operation, the less severe the disease. The results of this study revealed that DED after refractive surgery has a peak prevalence between 6 and 12 months and tends to improve over time. In this respect Shtein reported that post-LASIK DED usually peaks in first few months after surgery, and then symptoms begin to improve at 6–12 months after surgery in the vast majority of patients [4]. Pharmacologic therapy may be taken into consideration as part of comprehensive ocular surface management for individuals with persistent postoperative dry eye symptoms. Several anti-inflammatory and tear-stabilizing drugs can improve dry eye symptoms and ocular surface characteristics, according to a systematic review of topical ophthalmic medicines for keratoconjunctivitis sicca. These results offer crucial context for the care of patients who still have symptoms following refractive surgery, even though our study did not assess treatment outcomes [13]. Another survey concluded that the prevalence of DED after refractive surgery (LASIK) was 40% at 1 month, 20% at 3 months, and may drop up to 10% at 6 months [26]. Decreased corneal sensitivity after LASIK surgery had been observed in several studies; the sensitivity at 1 month postoperatively was significantly impaired compared to 6 months, representing corneal sensitivity similar to that in healthy individuals [28].

### Limitations

This study has several limitations. Its cross-sectional, questionnaire-based design limits causal inference and relies on self-reported data, which may introduce recall and reporting bias. The online survey distribution may also lead to selection bias and limit the generalizability of the findings. Additionally, the absence of preoperative ocular data makes it difficult to determine whether dry eye was pre-existing or surgery-induced with inability to verify surgery history. Some potential confounding factors, such as environmental and lifestyle influences, were not fully assessed. Finally, the small number of participants who underwent Femto-SMILE limited statistical power for this subgroup, and therefore no firm conclusions regarding this procedure can be drawn.

## Conclusion

Self-reported OSDI-defined dry eye symptoms were highly prevalent among respondents who had undergone refractive surgery in Saudi Arabia, with the highest rates observed following photorefractive keratectomy. The condition appears to be more pronounced in the early postoperative period and tends to improve over time. Geographic variation and surgical technique were identified as significant factors associated with OSDI-defined dry eye symptoms. The central region of Saudi Arabia and an operation time of <1 year were the most prevalent risk factors. To the best of our knowledge, this is the first study to address the prevalence and associated risk factors of OSDI-defined dry eye symptoms after refractive surgery in Saudi Arabia. These findings highlight the importance of patient counseling, careful surgical selection, and appropriate postoperative management to minimize the risk and severity of dry eye after refractive procedures.
