# Supplementary material for: Prevalence of self-reported dry eye symptoms after refractive surgery among Saudi adults: A cross-sectional survey
Source: PLoS One. 2026 Oct 29;21(10):e0361098. doi: 10.1371/journal.pone.0361098 (PMC13718936; doi:10.1371/journal.pone.0361098)
Supplement: Supplementary file 1 [file pone.0361098.s002.docx]

Dear Editor of PLOS One:

Thanks for revising my manuscript number# PONE-D-26-17620, titled “Prevalence and associated factors of dry eye after refractive surgery in the Saudi population”. Here are the answers of reviewer’s comments point by point.

Thanks for your comments about my manuscript, PONE-D-26-17620R1**,** Prevalence of self-reported dry eye symptoms after refractive surgery among Saudi adults: a cross-sectional survey.
Here are the answers of your comments:

1. In this instance it seems there may be acceptable restrictions in place that prevent the public sharing of your minimal data. However, in line with our goal of ensuring long-term data availability to all interested researchers, PLOS’ Data Policy states that authors cannot be the sole named individuals responsible for ensuring data access (<http://journals.plos.org/plosone/s/data-availability#loc-acceptable-data-sharing-methods>). Data requests to a non-author institutional point of contact, such as a data access or ethics committee, helps guarantee long term stability and availability of data. Providing interested researchers with a durable point of contact ensures data will be accessible even if an author changes email addresses, institutions, or becomes unavailable to answer requests. Before we proceed with your manuscript, please also provide non-author contact information (phone/email/hyperlink) for a data access committee, ethics committee, or other institutional body to which data requests may be sent. If no institutional body is available to respond to requests for your minimal data, please consider if there any institutional representatives who did not collaborate in the study, and are not listed as authors on the manuscript, who would be able to hold the data and respond to external requests for data access? If so, please provide their contact information (i.e., email address). Please also provide details on how you will ensure persistent or long-term data storage and availability.

**Response:** Thank you for this clarification. We have revised the Data Availability Statement to provide a non-author institutional point of contact for requests for access to the minimal dataset. We have also added information regarding the institutional arrangements for long-term storage and availability of the data. The corresponding-author-only data-access statement has been removed.

### The defensible institutional

**Institution:** King Abdulaziz University

**Institutional body:** Deanship of Scientific Research

**Email:** [dsrw@kau.edu.sa](mailto:dsrw@kau.edu.sa)

**Telephone:** **+966 12 695 0162**

**Official webpage:** [KAU My Research Portal / Deanship of Scientific Research contact page](https://kau.edu.sa/en/page/my-research-portal?utm_source=chatgpt.com)

KAU officially lists this email and telephone number for communication with the Deanship of Scientific Research. The Deanship is also officially described as the university body responsible for organizing, supporting, and enhancing scientific research.

**Data Availability Statement:** The minimal dataset underlying the findings of this study is available upon reasonable request through the Deanship of Scientific Research, King Abdulaziz University. Data access requests may be submitted to **dsrw@kau.edu.sa**. The dataset will be securely retained in accordance with King Abdulaziz University institutional policies, and the designated institutional body will facilitate continued access to the data for legitimate research purposes, subject to applicable ethical and data-protection requirements.

2. Please amend the title either on the online submission form or in the manuscript so that they are identical.

**Response:** Thank you. We have amended the manuscript title to exactly match the title entered in the online submission system. The title is now according to reviewers comments: **“**Prevalence of self-reported dry eye symptoms after refractive surgery among Saudi adults: a cross-sectional survey.”

Response to reviewers comments

| Queries | Answers |
| --- | --- |
| Reviewer 1 |  |
| First, The conclusions should be moderated because this is a cross-sectional online survey. The manuscript should consistently refer to "self-reported OSDI-defined dry eye symptoms after refractive surgery" rather than clinically confirmed dry eye disease or incidence. | We appreciate the reviewer's insightful feedback. We concur that OSDI assesses symptoms rather than clinically verified dry eye illness and that our cross-sectional questionnaire-based study cannot determine incidence or cause. Because of this, we have changed the wording used throughout the text to "self-reported OSDI-defined dry eye symptoms after refractive surgery" as applicable. Additionally, we changed the findings to prevent causal interpretation and substituted "prevalence" or "prevalence of symptoms" for "incidence" where appropriate. |
| Second, the sampling strategy creates serious risk of selection bias and should be described much more transparently. The questionnaire was distributed through social media platforms, and the manuscript reports a response rate of 100%, which is implausible for an open online survey unless the denominator was defined only as completed questionnaires. The authors must clarify how many individuals received, opened, started, and completed the survey, whether duplicate responses were prevented, whether IP or device restrictions were used, and how participants’ history and type of refractive surgery were verified. A self-selected online sample is likely to overrepresent symptomatic individuals, which may partly explain the extremely high 89.1% OSDI-defined prevalence and the 69.5% severe category. This limitation must be emphasized more strongly. | We value this significant finding. The sampling mechanism has been made clear. The study represents a convenience sample rather than a representative national sample because social media was used for recruitment. We made it clear that the stated 100% response rate only applies to completed surveys; it does not apply to everyone who saw the survey invitation. Additionally, we have noted that no clinical verification or medical records were available, and that individuals self-reported their history of refractive surgery. Additionally, selection bias has recently been highlighted as a significant drawback. |
| Third, the statistical analysis requires clearer and more rigorous presentation. The methods state that logistic regression was used, but the results mainly present odds ratios without sufficiently clarifying whether these were univariable or multivariable models. The authors should specify all covariates entered into the model, justify the reference categories, and provide a full regression table including age, sex, region, smoking, chronic disease, surgery type, and postoperative duration. This is particularly important because surgery type and time since surgery may be confounded by age, indication, surgical era, preoperative dry eye risk, and selection of procedure. The very small Femto-SMILE group, with only 16 participants, also prevents reliable conclusions about SMILE-related dry eye and should not be overinterpreted. | We are grateful to the reviewer. We have made it clear that a multivariable logistic regression model produced the presented odds ratios. All of the variables used in the model are now listed in the Methods section, and Table 2 has been updated to show adjusted odds ratios. Additionally, we recognize that the Femto-SMILE subgroup was quite tiny, thus findings on this process should be read with caution. |
| Fourth, the interpretation of geographic and environmental effects is too speculative. The authors suggest that living in the Eastern Province, a more humid area, may reduce postoperative dry eye risk, and report lower odds compared with the central region. However, the study did not measure actual humidity exposure, air conditioning use, occupational environment, screen time, outdoor exposure, workplace conditions, city-level climate, or seasonal variation. Regional differences may also reflect recruitment bias, socioeconomic differences, clinic or surgeon factors, postoperative care, or differences in procedure distribution. The authors should revise this section to state that regional association was observed, but environmental humidity remains an untested hypothesis rather than a demonstrated protective factor. | We concur. In order to avoid attributing regional variations to humidity, we have updated the discussion. Humidity is merely given as a potential hypothesis that was not directly tested, whereas the observed correlation is now reported as an observational finding. |
| Fifth, several reporting issues must be corrected to meet PLOS ONE standards. The ethics statement reports approval from the Unit of Biomedical Ethical Research Committee of King Abdulaziz University with reference number 1722, dated January 25, 2022, but the manuscript inconsistently states verbal consent in one section and written informed consent on the questionnaire page in another. The authors must clarify whether consent was written, electronic, or implied by questionnaire completion. In addition, the data availability statement “Data available with the corresponding author” is not sufficient for PLOS ONE unless there are legal or ethical restrictions. The authors should deposit the minimal anonymized dataset in a public repository or provide a specific ethical/legal justification for restricted access. These are not optional technical issues; they directly affect transparency and publication compliance. | We are grateful to the reviewer. The informed consent statement has been updated to reflect the electronic written consent that was acquired prior to the completion of the questionnaire. In accordance with PLOS ONE guidelines, the Data Availability Statement has also been updated. |
| Sixth, the manuscript would benefit from a more comprehensive contextualization within the current dry eye disease and refractive-surgery treatment landscape. Several relevant systematic reviews and meta-analyses addressing post-refractive dry eye, dry eye pharmacologic management, punctal occlusion strategies, and meibomian gland dysfunction should be cited to strengthen the background and discussion. The following listed 4 studies are very helpful to consider for citation: 1. PMID: 40778870; doi: 10.3928/1081597X-20250506-07, because it directly compares dry eye outcomes after keratorefractive lenticule extraction surgery and LASIK and is highly relevant to the discussion of procedure-specific postoperative dry eye risk; 2. PMID: 40679590; doi: 10.1007/s00210-025-04454-8, because it systematically evaluates topical ophthalmic drugs for keratoconjunctivitis sicca and would help contextualize treatment options for patients with persistent postoperative dry eye; 3. PMID: 40393913; doi: 10.1016/j.clae.2025.102438, because it evaluates punctal plugs for dry eye disease and is relevant to tear-conservation strategies after refractive surgery; and 4. PMID: 40360035; doi: 10.1016/j.pdpdt.2025.104630, because it compares LipiFlow and intense pulsed light for meibomian gland dysfunction, a key contributor to evaporative dry eye that may coexist with or worsen post-refractive symptoms. These references should be cited where they genuinely strengthen the discussion and should not be inserted superficially. | We appreciate the reviewer recommending these new sources. These papers are included in the Introduction and Discussion to support the interpretation of procedure-specific dry eye results and current care options, such as topical medication, punctal occlusion, and meibomian gland dysfunction treatment. |
| The manuscript also needs language and terminology correction. The abstract states that dry eye disease is “its most common side effects,” which should be corrected to “one of its most common side effects.” The authors should standardize “DED,” “dry eye disease,” “dry eye symptoms,” “postoperative DED,” “prevalence,” and “incidence.” The term “refractory surgery” appears in the methods and should be corrected to “refractive surgery.” The manuscript should also clarify whether LASIK and FS-LASIK were combined because of clinical similarity, sample-size limitations, or data-collection constraints. Figures and tables should include exact denominators and percentages, and Table 2 should clearly indicate adjusted versus unadjusted odds ratios. | We are grateful for the thorough linguistic review. The manuscript has been thoroughly edited in terms of language. Grammar mistakes were fixed, terminology was standardized throughout the book, "refractory surgery" was changed to "refractive surgery," the rationale behind the combination of LASIK and FS-LASIK was explained, and tables and figures were updated for clarity. |
| **Reviewer- 2** |  |
| 1. The authors should substantially revise the interpretation of “prevalence.” Because the study used an online, self-administered convenience survey distributed through social media, the sample is unlikely to represent all refractive surgery patients in Saudi Arabia. The reported 89.1% prevalence may reflect selection bias, especially if symptomatic patients were more likely to respond. The conclusions should be reframed as prevalence of self-reported dry eye symptoms among survey respondents. | We concur. Instead of describing the results as the prevalence across all Saudi Arabian patients undergoing refractive surgery, the paper has been updated to reflect the prevalence of self-reported dry eye complaints among survey respondents. |
| 2. The diagnosis of dry eye relies entirely on OSDI without objective clinical tests such as tear breakup time, Schirmer test, corneal staining, meibomian gland assessment, or ophthalmic examination. The authors should avoid equating OSDI-defined symptoms with clinically confirmed DED, or clearly justify this approach. | We concur. The text now makes it very clear that OSDI does not provide a clinical diagnosis of dry eye illness; rather, it measures symptoms. Both the Methods and Discussion have highlighted this restriction. |
| 3. The absence of preoperative dry eye status is a major limitation. Without baseline ocular surface data, it is impossible to determine whether symptoms were caused by refractive surgery or existed before surgery. This limitation should be emphasized, and causal language such as “after surgery caused DED” should be avoided. | We concur. One significant issue that has recently been highlighted is the lack of preoperative ocular surface evaluation. We have eliminated any language that implied dry eye condition was brought on by refractive surgery. |
| 4. The statistical analysis requires clarification. It is unclear whether the logistic regression was univariable or multivariable. The authors should provide a fully adjusted model including age, sex, region, smoking, chronic disease, surgery type, and time since surgery. They should also address sparse data problems, especially the very small Femto-SMILE group and the >50 years age groups. | We are grateful to the reviewer. Multivariable logistic regression was carried out utilizing age, sex, smoking status, chronic disease, geographic region, surgery type, and postoperative duration, according to the statistical procedures that have been clarified. We also recognize that estimations should be regarded with caution due to the small size of the Femto-SMILE and >50-year groups. |
| 5. The comparison among PRK, LASIK/FS-LASIK, and SMILE should be interpreted cautiously. Surgical groups are imbalanced, and important confounders such as preoperative refractive error, ablation depth, ocular surface treatment, surgeon/center, postoperative medication, and preoperative contact lens history were not available. | We concur. Important factors, such as preoperative refractive error, ablation depth, preoperative dry eye status, contact lens wear, surgeon, postoperative drugs, and ocular surface treatment, were not provided and may have complicated the observed relationships, as the Discussion now admits. |

Please accept my regards

**Corresponding author**

Abubakr S Alzwaihri

Department of Ophthalmology,

Jeddah Eye Hospital, Jeddah, Saudi Arabia

Phone: +966538416546

Email: abubakr.alzwaihri@gmail.com

https://orcid.org/0000-0001-9057-3707
