# Supplementary material for: Prevalence of self-reported dry eye symptoms after refractive surgery among Saudi adults: A cross-sectional survey
Source: PLoS One. 2026 Oct 29;21(10):e0361098. doi: 10.1371/journal.pone.0361098 (PMC13718936; doi:10.1371/journal.pone.0361098)
Supplement: Peer review history

# Peer review: Decision Letter 0

**DOI:** 10.1371/journal.pone.0361098.r001

**Authors:** Rajiv R. Mohan

29 Jun 2026

Dear Dr. Alzwaihri,

Thank you for submitting your manuscript to PLOS ONE. After careful consideration, we feel that it has merit but does not fully meet PLOS ONE’s publication criteria as it currently stands. Therefore, we invite you to submit a revised version of the manuscript that addresses the points raised during the review process.

Please submit your revised manuscript by Aug 13 2026 11:59PM. If you will need more time than this to complete your revisions, please reply to this message or contact the journal office at plosone@plos.org. When you're ready to submit your revision, log on to https://www.editorialmanager.com/pone/ and select the 'Submissions Needing Revision' folder to locate your manuscript file.

- A letter that responds to each point raised by the academic editor and reviewer(s). You should upload this letter as a separate file labeled 'Response to Reviewers'.
- A marked-up copy of your manuscript that highlights changes made to the original version. You should upload this as a separate file labeled 'Revised Manuscript with Track Changes'.
- An unmarked version of your revised paper without tracked changes. You should upload this as a separate file labeled 'Manuscript'.

If you would like to make changes to your financial disclosure, please include your updated statement in your cover letter. Guidelines for resubmitting your figure files are available below the reviewer comments at the end of this letter.

If applicable, we recommend that you deposit your laboratory protocols in protocols.io to enhance the reproducibility of your results. Protocols.io assigns your protocol its own identifier (DOI) so that it can be cited independently in the future. For instructions see: https://journals.plos.org/plosone/s/submission-guidelines#loc-laboratory-protocols. Additionally, PLOS ONE offers an option for publishing peer-reviewed Lab Protocol articles, which describe protocols hosted on protocols.io. Read more information on sharing protocols at https://plos.org/protocols?utm_medium=editorial-email&utm_source=authorletters&utm_campaign=protocols.

As the corresponding author, your ORCID iD is verified in the submission system and will appear in the published article. PLOS supports the use of ORCID, and we encourage all coauthors to register for an ORCID iD and use it as well. Please encourage your coauthors to verify their ORCID iD within the submission system before final acceptance, as unverified ORCID iDs will not appear in the published article. *Only*  the individual author can complete the verification step; PLOS staff *cannot*  verify ORCID iDs on behalf of authors.

We look forward to receiving your revised manuscript.

Kind regards,

Rajiv R. Mohan, Ph.D.

Academic Editor

PLOS One

Journal Requirements:

When submitting your revision, we need you to address these additional requirements.

1. Please ensure that your manuscript meets PLOS ONE's style requirements, including those for file naming. The PLOS ONE style templates can be found at

https://journals.plos.org/plosone/s/file?id=wjVg/PLOSOne_formatting_sample_main_body.pdf and

https://journals.plos.org/plosone/s/file?id=ba62/PLOSOne_formatting_sample_title_authors_affiliations.pdf.

2. In the ethics statement in the Methods, you have specified that verbal consent was obtained. Please provide additional details regarding how this consent was documented and witnessed, and state whether this was approved by the IRB.

3. In the online submission form you indicate that your data is not available for proprietary reasons and have provided a contact point for accessing this data. Please note that your current contact point is a co-author on this manuscript. According to our Data Policy, the contact point must not be an author on the manuscript and must be an institutional contact, ideally not an individual. Please revise your data statement to a non-author institutional point of contact, such as a data access or ethics committee, and send this to us via return email. Please also include contact information for the third party organization, and please include the full citation of where the data can be found.

4. Your ethics statement should only appear in the Methods section of your manuscript. If your ethics statement is written in any section besides the Methods, please delete it from any other section.

5. Please include captions for your Supporting Information files at the end of your manuscript, and update any in-text citations to match accordingly. Please see our Supporting Information guidelines for more information: http://journals.plos.org/plosone/s/supporting-information.

6. If the reviewer comments include a recommendation to cite specific previously published works, please review and evaluate these publications to determine whether they are relevant and should be cited. There is no requirement to cite these works unless the editor has indicated otherwise.

7. Please review your reference list to ensure that it is complete and correct. If you have cited papers that have been retracted, please include the rationale for doing so in the manuscript text, or remove these references and replace them with relevant current references. Any changes to the reference list should be mentioned in the rebuttal letter that accompanies your revised manuscript. If you need to cite a retracted article, indicate the article’s retracted status in the References list and also include a citation and full reference for the retraction notice.

Additional Editor Comments (if provided):

Dear authors,

Your manuscript has completed peer-review process and comments are appended below. You will see that the reviewers though find research has merit but have many concerns. Hence, I recommend adequate addressing of all concerns through additional experimental data and evidence from literature. Thank you

Reviewer-1

The manuscript entitled “Prevalence and associated factors of dry eye after refractive surgery in the Saudi population” addresses a clinically relevant topic for PLOS ONE. The authors conducted a questionnaire-based survey of 439 participants in Saudi Arabia who had undergone refractive surgery and assessed dry eye disease using the Ocular Surface Disease Index. The manuscript reports that 391 participants, or 89.1%, had dry eye disease after surgery, with 69.5% classified as severe, 11.4% as moderate, and 8.2% as mild. The study further reports that PRK was performed in 201 participants, LASIK or FS-LASIK in 222 participants, and Femto-SMILE in only 16 participants, with lower odds of dry eye among residents of the Eastern Province, lower odds after LASIK or FS-LASIK compared with PRK, and lower odds among patients with longer postoperative duration. The topic is publishable and regionally useful, but the current version still requires minor revision because several methodological, statistical, interpretive, and reporting issues remain insufficiently controlled.

First, the authors must moderate the central conclusion because the design does not allow estimation of true postoperative incidence or causal risk. This is a descriptive, cross-sectional, self-administered online survey without preoperative OSDI scores, clinical ocular-surface examination, tear break-up time, Schirmer testing, corneal staining, meibomian gland assessment, or objective confirmation of dry eye disease. Therefore, the manuscript should consistently refer to “self-reported OSDI-defined dry eye symptoms after refractive surgery” rather than definitive clinical dry eye disease or incidence. The phrase “PRK was associated with a higher incidence of dry eye” is not appropriate because the study measured prevalence at variable postoperative intervals, not incident disease after surgery.

Second, the sampling strategy creates serious risk of selection bias and should be described much more transparently. The questionnaire was distributed through social media platforms, and the manuscript reports a response rate of 100%, which is implausible for an open online survey unless the denominator was defined only as completed questionnaires. The authors must clarify how many individuals received, opened, started, and completed the survey, whether duplicate responses were prevented, whether IP or device restrictions were used, and how participants’ history and type of refractive surgery were verified. A self-selected online sample is likely to overrepresent symptomatic individuals, which may partly explain the extremely high 89.1% OSDI-defined prevalence and the 69.5% severe category. This limitation must be emphasized more strongly.

Third, the statistical analysis requires clearer and more rigorous presentation. The methods state that logistic regression was used, but the results mainly present odds ratios without sufficiently clarifying whether these were univariable or multivariable models. The authors should specify all covariates entered into the model, justify the reference categories, and provide a full regression table including age, sex, region, smoking, chronic disease, surgery type, and postoperative duration. This is particularly important because surgery type and time since surgery may be confounded by age, indication, surgical era, preoperative dry eye risk, and selection of procedure. The very small Femto-SMILE group, with only 16 participants, also prevents reliable conclusions about SMILE-related dry eye and should not be overinterpreted.

Fourth, the interpretation of geographic and environmental effects is too speculative. The authors suggest that living in the Eastern Province, a more humid area, may reduce postoperative dry eye risk, and report lower odds compared with the central region. However, the study did not measure actual humidity exposure, air conditioning use, occupational environment, screen time, outdoor exposure, workplace conditions, city-level climate, or seasonal variation. Regional differences may also reflect recruitment bias, socioeconomic differences, clinic or surgeon factors, postoperative care, or differences in procedure distribution. The authors should revise this section to state that regional association was observed, but environmental humidity remains an untested hypothesis rather than a demonstrated protective factor.

Fifth, several reporting issues must be corrected to meet PLOS ONE standards. The ethics statement reports approval from the Unit of Biomedical Ethical Research Committee of King Abdulaziz University with reference number 1722, dated January 25, 2022, but the manuscript inconsistently states verbal consent in one section and written informed consent on the questionnaire page in another. The authors must clarify whether consent was written, electronic, or implied by questionnaire completion. In addition, the data availability statement “Data available with the corresponding author” is not sufficient for PLOS ONE unless there are legal or ethical restrictions. The authors should deposit the minimal anonymized dataset in a public repository or provide a specific ethical/legal justification for restricted access. These are not optional technical issues; they directly affect transparency and publication compliance.

Sixth, the manuscript would benefit from a more comprehensive contextualization within the current dry eye disease and refractive-surgery treatment landscape. Several relevant systematic reviews and meta-analyses addressing post-refractive dry eye, dry eye pharmacologic management, punctal occlusion strategies, and meibomian gland dysfunction should be cited to strengthen the background and discussion. The following listed 4 studies are very helpful to consider for citation: 1. PMID: 40778870; doi: 10.3928/1081597X-20250506-07, because it directly compares dry eye outcomes after keratorefractive lenticule extraction surgery and LASIK and is highly relevant to the discussion of procedure-specific postoperative dry eye risk; 2. PMID: 40679590; doi: 10.1007/s00210-025-04454-8, because it systematically evaluates topical ophthalmic drugs for keratoconjunctivitis sicca and would help contextualize treatment options for patients with persistent postoperative dry eye; 3. PMID: 40393913; doi: 10.1016/j.clae.2025.102438, because it evaluates punctal plugs for dry eye disease and is relevant to tear-conservation strategies after refractive surgery; and 4. PMID: 40360035; doi: 10.1016/j.pdpdt.2025.104630, because it compares LipiFlow and intense pulsed light for meibomian gland dysfunction, a key contributor to evaporative dry eye that may coexist with or worsen post-refractive symptoms. These references should be cited where they genuinely strengthen the discussion and should not be inserted superficially.

The manuscript also needs language and terminology correction. The abstract states that dry eye disease is “its most common side effects,” which should be corrected to “one of its most common side effects.” The authors should standardize “DED,” “dry eye disease,” “dry eye symptoms,” “postoperative DED,” “prevalence,” and “incidence.” The term “refractory surgery” appears in the methods and should be corrected to “refractive surgery.” The manuscript should also clarify whether LASIK and FS-LASIK were combined because of clinical similarity, sample-size limitations, or data-collection constraints. Figures and tables should include exact denominators and percentages, and Table 2 should clearly indicate adjusted versus unadjusted odds ratios.

Overall, this manuscript addresses a relevant regional question and provides potentially useful preliminary data on OSDI-defined dry eye symptoms after refractive surgery in Saudi Arabia. However, the authors must revise the manuscript to avoid causal and incidence language, clarify the online sampling method and response-rate denominator, present multivariable statistics transparently, moderate geographic and procedure-specific interpretations, correct the ethics and data availability statements, and improve language precision. I recommend minor revision. The manuscript can be accepted after minor revision if these issues are addressed carefully and consistently.

Reviewer-2

This manuscript investigates the prevalence and associated factors of dry eye symptoms after refractive surgery in Saudi Arabia using an online questionnaire and OSDI scoring. The topic is clinically relevant, and the sample size is reasonable. However, several methodological and reporting issues must be addressed before the manuscript can be considered scientifically sound.

1.The authors should substantially revise the interpretation of “prevalence.” Because the study used an online, self-administered convenience survey distributed through social media, the sample is unlikely to represent all refractive surgery patients in Saudi Arabia. The reported 89.1% prevalence may reflect selection bias, especially if symptomatic patients were more likely to respond. The conclusions should be reframed as prevalence of self-reported dry eye symptoms among survey respondents.

2.The diagnosis of dry eye relies entirely on OSDI without objective clinical tests such as tear breakup time, Schirmer test, corneal staining, meibomian gland assessment, or ophthalmic examination. The authors should avoid equating OSDI-defined symptoms with clinically confirmed DED, or clearly justify this approach.

3.The absence of preoperative dry eye status is a major limitation. Without baseline ocular surface data, it is impossible to determine whether symptoms were caused by refractive surgery or existed before surgery. This limitation should be emphasized, and causal language such as “after surgery caused DED” should be avoided.

4.The statistical analysis requires clarification. It is unclear whether the logistic regression was univariable or multivariable. The authors should provide a fully adjusted model including age, sex, region, smoking, chronic disease, surgery type, and time since surgery. They should also address sparse data problems, especially the very small Femto-SMILE group and the >50 years age group.

5.The comparison among PRK, LASIK/FS-LASIK, and SMILE should be interpreted cautiously. Surgical groups are imbalanced, and important confounders such as preoperative refractive error, ablation depth, ocular surface treatment, surgeon/center, postoperative medication, and preoperative contact lens history were not available.

[Note: HTML markup is below. Please do not edit.]

Reviewers' comments:

Reviewer's Responses to Questions

**Comments to the Author**

1. Is the manuscript technically sound, and do the data support the conclusions?

Reviewer #1: Yes

Reviewer #2: Partly

**********

2. Has the statistical analysis been performed appropriately and rigorously?

Reviewer #1: Yes

Reviewer #2: No

**********

3. Have the authors made all data underlying the findings in their manuscript fully available?

The PLOS Data policy

Reviewer #1: Yes

Reviewer #2: Yes

**********

4. Is the manuscript presented in an intelligible fashion and written in standard English?

Reviewer #1: Yes

Reviewer #2: Yes

**********

Reviewer #1: The manuscript entitled “Prevalence and associated factors of dry eye after refractive surgery in the Saudi population” addresses a clinically relevant topic for PLOS ONE. The authors conducted a questionnaire-based survey of 439 participants in Saudi Arabia who had undergone refractive surgery and assessed dry eye disease using the Ocular Surface Disease Index. The manuscript reports that 391 participants, or 89.1%, had dry eye disease after surgery, with 69.5% classified as severe, 11.4% as moderate, and 8.2% as mild. The study further reports that PRK was performed in 201 participants, LASIK or FS-LASIK in 222 participants, and Femto-SMILE in only 16 participants, with lower odds of dry eye among residents of the Eastern Province, lower odds after LASIK or FS-LASIK compared with PRK, and lower odds among patients with longer postoperative duration. The topic is publishable and regionally useful, but the current version still requires minor revision because several methodological, statistical, interpretive, and reporting issues remain insufficiently controlled.

First, the authors must moderate the central conclusion because the design does not allow estimation of true postoperative incidence or causal risk. This is a descriptive, cross-sectional, self-administered online survey without preoperative OSDI scores, clinical ocular-surface examination, tear break-up time, Schirmer testing, corneal staining, meibomian gland assessment, or objective confirmation of dry eye disease. Therefore, the manuscript should consistently refer to “self-reported OSDI-defined dry eye symptoms after refractive surgery” rather than definitive clinical dry eye disease or incidence. The phrase “PRK was associated with a higher incidence of dry eye” is not appropriate because the study measured prevalence at variable postoperative intervals, not incident disease after surgery.

Second, the sampling strategy creates serious risk of selection bias and should be described much more transparently. The questionnaire was distributed through social media platforms, and the manuscript reports a response rate of 100%, which is implausible for an open online survey unless the denominator was defined only as completed questionnaires. The authors must clarify how many individuals received, opened, started, and completed the survey, whether duplicate responses were prevented, whether IP or device restrictions were used, and how participants’ history and type of refractive surgery were verified. A self-selected online sample is likely to overrepresent symptomatic individuals, which may partly explain the extremely high 89.1% OSDI-defined prevalence and the 69.5% severe category. This limitation must be emphasized more strongly.

Third, the statistical analysis requires clearer and more rigorous presentation. The methods state that logistic regression was used, but the results mainly present odds ratios without sufficiently clarifying whether these were univariable or multivariable models. The authors should specify all covariates entered into the model, justify the reference categories, and provide a full regression table including age, sex, region, smoking, chronic disease, surgery type, and postoperative duration. This is particularly important because surgery type and time since surgery may be confounded by age, indication, surgical era, preoperative dry eye risk, and selection of procedure. The very small Femto-SMILE group, with only 16 participants, also prevents reliable conclusions about SMILE-related dry eye and should not be overinterpreted.

Fourth, the interpretation of geographic and environmental effects is too speculative. The authors suggest that living in the Eastern Province, a more humid area, may reduce postoperative dry eye risk, and report lower odds compared with the central region. However, the study did not measure actual humidity exposure, air conditioning use, occupational environment, screen time, outdoor exposure, workplace conditions, city-level climate, or seasonal variation. Regional differences may also reflect recruitment bias, socioeconomic differences, clinic or surgeon factors, postoperative care, or differences in procedure distribution. The authors should revise this section to state that regional association was observed, but environmental humidity remains an untested hypothesis rather than a demonstrated protective factor.

Fifth, several reporting issues must be corrected to meet PLOS ONE standards. The ethics statement reports approval from the Unit of Biomedical Ethical Research Committee of King Abdulaziz University with reference number 1722, dated January 25, 2022, but the manuscript inconsistently states verbal consent in one section and written informed consent on the questionnaire page in another. The authors must clarify whether consent was written, electronic, or implied by questionnaire completion. In addition, the data availability statement “Data available with the corresponding author” is not sufficient for PLOS ONE unless there are legal or ethical restrictions. The authors should deposit the minimal anonymized dataset in a public repository or provide a specific ethical/legal justification for restricted access. These are not optional technical issues; they directly affect transparency and publication compliance.

Sixth, the manuscript would benefit from a more comprehensive contextualization within the current dry eye disease and refractive-surgery treatment landscape. Several relevant systematic reviews and meta-analyses addressing post-refractive dry eye, dry eye pharmacologic management, punctal occlusion strategies, and meibomian gland dysfunction should be cited to strengthen the background and discussion. The following listed 4 studies are very helpful to consider for citation: 1. PMID: 40778870; doi: 10.3928/1081597X-20250506-07, because it directly compares dry eye outcomes after keratorefractive lenticule extraction surgery and LASIK and is highly relevant to the discussion of procedure-specific postoperative dry eye risk; 2. PMID: 40679590; doi: 10.1007/s00210-025-04454-8, because it systematically evaluates topical ophthalmic drugs for keratoconjunctivitis sicca and would help contextualize treatment options for patients with persistent postoperative dry eye; 3. PMID: 40393913; doi: 10.1016/j.clae.2025.102438, because it evaluates punctal plugs for dry eye disease and is relevant to tear-conservation strategies after refractive surgery; and 4. PMID: 40360035; doi: 10.1016/j.pdpdt.2025.104630, because it compares LipiFlow and intense pulsed light for meibomian gland dysfunction, a key contributor to evaporative dry eye that may coexist with or worsen post-refractive symptoms. These references should be cited where they genuinely strengthen the discussion and should not be inserted superficially.

The manuscript also needs language and terminology correction. The abstract states that dry eye disease is “its most common side effects,” which should be corrected to “one of its most common side effects.” The authors should standardize “DED,” “dry eye disease,” “dry eye symptoms,” “postoperative DED,” “prevalence,” and “incidence.” The term “refractory surgery” appears in the methods and should be corrected to “refractive surgery.” The manuscript should also clarify whether LASIK and FS-LASIK were combined because of clinical similarity, sample-size limitations, or data-collection constraints. Figures and tables should include exact denominators and percentages, and Table 2 should clearly indicate adjusted versus unadjusted odds ratios.

Overall, this manuscript addresses a relevant regional question and provides potentially useful preliminary data on OSDI-defined dry eye symptoms after refractive surgery in Saudi Arabia. However, the authors must revise the manuscript to avoid causal and incidence language, clarify the online sampling method and response-rate denominator, present multivariable statistics transparently, moderate geographic and procedure-specific interpretations, correct the ethics and data availability statements, and improve language precision. I recommend minor revision. The manuscript can be accepted after minor revision if these issues are addressed carefully and consistently.

Reviewer #2: This manuscript investigates the prevalence and associated factors of dry eye symptoms after refractive surgery in Saudi Arabia using an online questionnaire and OSDI scoring. The topic is clinically relevant, and the sample size is reasonable. However, several methodological and reporting issues must be addressed before the manuscript can be considered scientifically sound.

1.The authors should substantially revise the interpretation of “prevalence.” Because the study used an online, self-administered convenience survey distributed through social media, the sample is unlikely to represent all refractive surgery patients in Saudi Arabia. The reported 89.1% prevalence may reflect selection bias, especially if symptomatic patients were more likely to respond. The conclusions should be reframed as prevalence of self-reported dry eye symptoms among survey respondents.

2.The diagnosis of dry eye relies entirely on OSDI without objective clinical tests such as tear breakup time, Schirmer test, corneal staining, meibomian gland assessment, or ophthalmic examination. The authors should avoid equating OSDI-defined symptoms with clinically confirmed DED, or clearly justify this approach.

3.The absence of preoperative dry eye status is a major limitation. Without baseline ocular surface data, it is impossible to determine whether symptoms were caused by refractive surgery or existed before surgery. This limitation should be emphasized, and causal language such as “after surgery caused DED” should be avoided.

4.The statistical analysis requires clarification. It is unclear whether the logistic regression was univariable or multivariable. The authors should provide a fully adjusted model including age, sex, region, smoking, chronic disease, surgery type, and time since surgery. They should also address sparse data problems, especially the very small Femto-SMILE group and the >50 years age group.

5.The comparison among PRK, LASIK/FS-LASIK, and SMILE should be interpreted cautiously. Surgical groups are imbalanced, and important confounders such as preoperative refractive error, ablation depth, ocular surface treatment, surgeon/center, postoperative medication, and preoperative contact lens history were not available.

**********

what does this mean?). If published, this will include your full peer review and any attached files.

If you choose “no”, your identity will remain anonymous but your review may still be made public.

**Do you want your identity to be public for this peer review?**  For information about this choice, including consent withdrawal, please see our Privacy Policy

Reviewer #1: No

Reviewer #2: No

**********

[NOTE: If reviewer comments were submitted as an attachment file, they will be attached to this email and accessible via the submission site. Please log into your account, locate the manuscript record, and check for the action link "View Attachments". If this link does not appear, there are no attachment files.]

To ensure your figures meet our technical requirements, please review our figure guidelines: https://journals.plos.org/plosone/s/figures

You may also use PLOS’s free figure tool, NAAS, to help you prepare publication quality figures: https://journals.plos.org/plosone/s/figures#loc-tools-for-figure-preparation.

NAAS will assess whether your figures meet our technical requirements by comparing each figure against our figure specifications.

# Author comment: Author response to Decision Letter 1

**DOI:** 10.1371/journal.pone.0361098.r002

11 Sep 2026

Dear Editor of PLOS One:

Thanks for revising my manuscript number# PONE-D-26-17620, titled “Prevalence and associated factors of dry eye after refractive surgery in the Saudi population”. Here are the answers of reviewer’s comments point by point.

Thanks for your comments about my manuscript, PONE-D-26-17620R1, Prevalence of self-reported dry eye symptoms after refractive surgery among Saudi adults: a cross-sectional survey.

Here are the answers of your comments:

1. In this instance it seems there may be acceptable restrictions in place that prevent the public sharing of your minimal data. However, in line with our goal of ensuring long-term data availability to all interested researchers, PLOS’ Data Policy states that authors cannot be the sole named individuals responsible for ensuring data access (http://journals.plos.org/plosone/s/data-availability#loc-acceptable-data-sharing-methods). Data requests to a non-author institutional point of contact, such as a data access or ethics committee, helps guarantee long term stability and availability of data. Providing interested researchers with a durable point of contact ensures data will be accessible even if an author changes email addresses, institutions, or becomes unavailable to answer requests. Before we proceed with your manuscript, please also provide non-author contact information (phone/email/hyperlink) for a data access committee, ethics committee, or other institutional body to which data requests may be sent. If no institutional body is available to respond to requests for your minimal data, please consider if there any institutional representatives who did not collaborate in the study, and are not listed as authors on the manuscript, who would be able to hold the data and respond to external requests for data access? If so, please provide their contact information (i.e., email address). Please also provide details on how you will ensure persistent or long-term data storage and availability.

Response: Thank you for this clarification. We have revised the Data Availability Statement to provide a non-author institutional point of contact for requests for access to the minimal dataset. We have also added information regarding the institutional arrangements for long-term storage and availability of the data. The corresponding-author-only data-access statement has been removed.

The defensible institutional

Institution: King Abdulaziz University

Institutional body: Deanship of Scientific Research

Email: dsrw@kau.edu.sa

Telephone: +966 12 695 0162

Official webpage: KAU My Research Portal / Deanship of Scientific Research contact page

KAU officially lists this email and telephone number for communication with the Deanship of Scientific Research. The Deanship is also officially described as the university body responsible for organizing, supporting, and enhancing scientific research.

Data Availability Statement: The minimal dataset underlying the findings of this study is available upon reasonable request through the Deanship of Scientific Research, King Abdulaziz University. Data access requests may be submitted to dsrw@kau.edu.sa. The dataset will be securely retained in accordance with King Abdulaziz University institutional policies, and the designated institutional body will facilitate continued access to the data for legitimate research purposes, subject to applicable ethical and data-protection requirements.

2. Please amend the title either on the online submission form or in the manuscript so that they are identical.

Response: Thank you. We have amended the manuscript title to exactly match the title entered in the online submission system. The title is now according to reviewers comments: “Prevalence of self-reported dry eye symptoms after refractive surgery among Saudi adults: a cross-sectional survey.”

Response to reviewers comments

Queries Answers

Reviewer 1

First, The conclusions should be moderated because this is a cross-sectional online survey. The manuscript should consistently refer to "self-reported OSDI-defined dry eye symptoms after refractive surgery" rather than clinically confirmed dry eye disease or incidence. We appreciate the reviewer's insightful feedback. We concur that OSDI assesses symptoms rather than clinically verified dry eye illness and that our cross-sectional questionnaire-based study cannot determine incidence or cause. Because of this, we have changed the wording used throughout the text to "self-reported OSDI-defined dry eye symptoms after refractive surgery" as applicable. Additionally, we changed the findings to prevent causal interpretation and substituted "prevalence" or "prevalence of symptoms" for "incidence" where appropriate.

Second, the sampling strategy creates serious risk of selection bias and should be described much more transparently. The questionnaire was distributed through social media platforms, and the manuscript reports a response rate of 100%, which is implausible for an open online survey unless the denominator was defined only as completed questionnaires. The authors must clarify how many individuals received, opened, started, and completed the survey, whether duplicate responses were prevented, whether IP or device restrictions were used, and how participants’ history and type of refractive surgery were verified. A self-selected online sample is likely to overrepresent symptomatic individuals, which may partly explain the extremely high 89.1% OSDI-defined prevalence and the 69.5% severe category. This limitation must be emphasized more strongly.

We value this significant finding. The sampling mechanism has been made clear. The study represents a convenience sample rather than a representative national sample because social media was used for recruitment. We made it clear that the stated 100% response rate only applies to completed surveys; it does not apply to everyone who saw the survey invitation. Additionally, we have noted that no clinical verification or medical records were available, and that individuals self-reported their history of refractive surgery. Additionally, selection bias has recently been highlighted as a significant drawback.

Third, the statistical analysis requires clearer and more rigorous presentation. The methods state that logistic regression was used, but the results mainly present odds ratios without sufficiently clarifying whether these were univariable or multivariable models. The authors should specify all covariates entered into the model, justify the reference categories, and provide a full regression table including age, sex, region, smoking, chronic disease, surgery type, and postoperative duration. This is particularly important because surgery type and time since surgery may be confounded by age, indication, surgical era, preoperative dry eye risk, and selection of procedure. The very small Femto-SMILE group, with only 16 participants, also prevents reliable conclusions about SMILE-related dry eye and should not be overinterpreted.

We are grateful to the reviewer. We have made it clear that a multivariable logistic regression model produced the presented odds ratios. All of the variables used in the model are now listed in the Methods section, and Table 2 has been updated to show adjusted odds ratios. Additionally, we recognize that the Femto-SMILE subgroup was quite tiny, thus findings on this process should be read with caution.

Fourth, the interpretation of geographic and environmental effects is too speculative. The authors suggest that living in the Eastern Province, a more humid area, may reduce postoperative dry eye risk, and report lower odds compared with the central region. However, the study did not measure actual humidity exposure, air conditioning use, occupational environment, screen time, outdoor exposure, workplace conditions, city-level climate, or seasonal variation. Regional differences may also reflect recruitment bias, socioeconomic differences, clinic or surgeon factors, postoperative care, or differences in procedure distribution. The authors should revise this section to state that regional association was observed, but environmental humidity remains an untested hypothesis rather than a demonstrated protective factor. We concur. In order to avoid attributing regional variations to humidity, we have updated the discussion. Humidity is merely given as a potential hypothesis that was not directly tested, whereas the observed correlation is now reported as an observational finding.

Fifth, several reporting issues must be corrected to meet PLOS ONE standards. The ethics statement reports approval from the Unit of Biomedical Ethical Research Committee of King Abdulaziz University with reference number 1722, dated January 25, 2022, but the manuscript inconsistently states verbal consent in one section and written informed consent on the questionnaire page in another. The authors must clarify whether consent was written, electronic, or implied by questionnaire completion. In addition, the data availability statement “Data available with the corresponding author” is not sufficient for PLOS ONE unless there are legal or ethical restrictions. The authors should deposit the minimal anonymized dataset in a public repository or provide a specific ethical/legal justification for restricted access. These are not optional technical issues; they directly affect transparency and publication compliance. We are grateful to the reviewer. The informed consent statement has been updated to reflect the electronic written consent that was acquired prior to the completion of the questionnaire. In accordance with PLOS ONE guidelines, the Data Availability Statement has also been updated.

Sixth, the manuscript would benefit from a more comprehensive contextualization within the current dry eye disease and refractive-surgery treatment landscape. Several relevant systematic reviews and meta-analyses addressing post-refractive dry eye, dry eye pharmacologic management, punctal occlusion strategies, and meibomian gland dysfunction should be cited to strengthen the background and discussion. The following listed 4 studies are very helpful to consider for citation: 1. PMID: 40778870; doi: 10.3928/1081597X-20250506-07, because it directly compares dry eye outcomes after keratorefractive lenticule extraction surgery and LASIK and is highly relevant to the discussion of procedure-specific postoperative dry eye risk; 2. PMID: 40679590; doi: 10.1007/s00210-025-04454-8, because it systematically evaluates topical ophthalmic drugs for keratoconjunctivitis sicca and would help contextualize treatment options for patients with persistent postoperative dry eye; 3. PMID: 40393913; doi: 10.1016/j.clae.2025.102438, because it evaluates punctal plugs for dry eye disease and is relevant to tear-conservation strategies after refractive surgery; and 4. PMID: 40360035; doi: 10.1016/j.pdpdt.2025.104630, because it compares LipiFlow and intense pulsed light for meibomian gland dysfunction, a key contributor to evaporative dry eye that may coexist with or worsen post-refractive symptoms. These references should be cited where they genuinely strengthen the discussion and should not be inserted superficially. We appreciate the reviewer recommending these new sources. These papers are included in the Introduction and Discussion to support the interpretation of procedure-specific dry eye results and current care options, such as topical medication, punctal occlusion, and meibomian gland dysfunction treatment.

The manuscript also needs language and terminology correction. The abstract states that dry eye disease is “its most common side effects,” which should be corrected to “one of its most common side effects.” The authors should standardize “DED,” “dry eye disease,” “dry eye symptoms,” “postoperative DED,” “prevalence,” and “incidence.” The term “refractory surgery” appears in the methods and should be corrected to “refractive surgery.” The manuscript should also clarify whether LASIK and FS-LASIK were combined because of clinical similarity, sample-size limitations, or data-collection constraints. Figures and tables should include exact denominators and percentages, and Table 2 should clearly indicate adjusted versus unadjusted odds ratios. We are grateful for the thorough linguistic review. The manuscript has been thoroughly edited in terms of language. Grammar mistakes were fixed, terminology was standardized throughout the book, "refractory surgery" was changed to "refractive surgery," the rationale behind the combination of LASIK and FS-LASIK was explained, and tables and figures were updated for clarity.

Reviewer- 2

1. The authors should substantially revise the interpretation of “prevalence.” Because the study used an online, self-administered convenience survey distributed through social media, the sample is unlikely to represent all refractive surgery patients in Saudi Arabia. The reported 89.1% prevalence may reflect selection bias, especially if symptomatic patients were more likely to respond. The conclusions should be reframed as prevalence of self-reported dry eye symptoms among survey respondents. We concur. Instead of describing the results as the prevalence across all Saudi Arabian patients undergoing refractive surgery, the paper has been updated to reflect the prevalence of self-reported dry eye complaints among survey respondents.

2. The diagnosis of dry eye relies entirely on OSDI without objective clinical tests such as tear breakup time, Schirmer test, corneal staining, meibomian gland assessment, or ophthalmic examination. The authors should avoid equating OSDI-defined symptoms with clinically confirmed DED, or clearly justify this approach. We concur. The text now makes it very clear that OSDI does not provide a clinical diagnosis of dry eye illness; rather, it measures symptoms. Both the Methods and Discussion have highlighted this restriction.

3. The absence of preoperative dry eye status is a major limitation. Without baseline ocular surface data, it is impossible to determine whether symptoms were caused by refractive surgery or existed before surgery. This limitation should be emphasized, and causal language such as “after surgery caused DED” should be avoided. We concur. One significant issue that has recently been highlighted is the lack of preoperative ocular surface evaluation. We have eliminated any language that implied dry eye condition was brought on by refractive surgery.

4. The statistical analysis requires clarification. It is unclear whether the logistic regression was univariable or multivariable. The authors should provide a fully adjusted model including age, sex, region, smoking, chronic disease, surgery type, and time since surgery. They should also address sparse data problems, especially the very small Femto-SMILE group and the >50 years age groups. We are grateful to the reviewer. Multivariable logistic regression was carried out utilizing age, sex, smoking status, chronic disease, geographic region, surgery type, and postoperative duration, according to the statistical procedures that have been clarified. We also recognize that estimations should be regarded with caution due to the small size of the Femto-SMILE and >50-year groups.

5. The comparison among PRK, LASIK/FS-LASIK, and SMILE should be interpreted cautiously. Surgical groups are imbalanced, and important confounders such as preoperative refractive error, ablation depth, ocular surface treatment, surgeon/center, postoperative medication, and preoperative contact lens history were not available. We concur. Important factors, such as preoperative refractive error, ablation depth, preoperative dry eye status, contact lens wear, surgeon, postoperative drugs, and ocular surface treatment, were not provided and may have complicated the observed relationships, as the Discussion now admits.

Attachment Submitted filename: Answers of reviewers comments.docx

# Editor report: Decision Letter 1

**DOI:** 10.1371/journal.pone.0361098.r003

**Authors:** Rajiv R. Mohan

7 Oct 2026

Prevalence of self-reported dry eye symptoms after refractive surgery among Saudi adults: a cross-sectional survey

PONE-D-26-17620R1

Dear Dr. %Alzwaihri%,

We’re pleased to inform you that your manuscript has been judged scientifically suitable for publication and will be formally accepted for publication once it meets all outstanding technical requirements.

Within one week, you’ll receive an e-mail detailing the required amendments. When these have been addressed, you’ll receive a formal acceptance letter and your manuscript will be scheduled for publication.

An invoice will be generated when your article is formally accepted. Please note, if your institution has a publishing partnership with PLOS and your article meets the relevant criteria, all or part of your publication costs will be covered. Please make sure your user information is up-to-date by logging into Editorial Manager at Editorial Manager® and clicking the ‘Update My Information' link at the top of the page. For questions related to billing, please contact billing support.

If your institution or institutions have a press office, please notify them about your upcoming paper to help maximize its impact. If they’ll be preparing press materials, please inform our press team as soon as possible -- no later than 48 hours after receiving the formal acceptance. Your manuscript will remain under strict press embargo until 2 pm Eastern Time on the date of publication. For more information, please contact onepress@plos.org.

Kind regards,

Rajiv R. Mohan, Ph.D.

Academic Editor

PLOS One

Additional Editor Comments (optional):

Dear authors,

I am happy to inform that your revisions/changes adequately addressed all concerns. The manuscript is accepted for publication. Congratulations!

Reviewers' comments:

# Editor report: Acceptance letter

**DOI:** 10.1371/journal.pone.0361098.r004

**Authors:** Rajiv R. Mohan

PONE-D-26-17620R1

PLOS One

Dear Dr. Alzwaihri,

I'm pleased to inform you that your manuscript has been deemed suitable for publication in PLOS One. Congratulations! Your manuscript is now being handed over to our production team.

At this stage, our production department will prepare your paper for publication. This includes ensuring the following:

* All references, tables, and figures are properly cited

* All relevant supporting information is included in the manuscript submission,

* There are no issues that prevent the paper from being properly typeset

You will receive further instructions from the production team, including instructions on how to review your proof when it is ready. Please keep in mind that we are working through a large volume of accepted articles, so please give us a few days to review your paper and let you know the next and final steps.

Lastly, if your institution or institutions have a press office, please let them know about your upcoming paper now to help maximize its impact. If they'll be preparing press materials, please inform our press team within the next 48 hours. Your manuscript will remain under strict press embargo until 2 pm Eastern Time on the date of publication. For more information, please contact onepress@plos.org.

You will receive an invoice from PLOS for your publication fee after your manuscript has reached the completed accept phase. If you receive an email requesting payment before acceptance or for any other service, this may be a phishing scheme. Learn how to identify phishing emails and protect your accounts at https://explore.plos.org/phishing.

If we can help with anything else, please email us at customercare@plos.org.

Thank you for submitting your work to PLOS One and supporting open access.

Kind regards,

PLOS One Editorial Office Staff

on behalf of

Dr. Rajiv R. Mohan

Academic Editor

PLOS One
